# Synthesis and characterization of conductive flexible cellulose carbon nanohorn sheets for human tissue applications

**DOI:** 10.1186/s40824-020-00194-3

**Published:** 2020-10-23

**Authors:** Karthik Paneer Selvam, Taichi Nagahata, Kosuke Kato, Mayuko Koreishi, Toshiyuki Nakamura, Yoshimasa Nakamura, Takeshi Nishikawa, Ayano Satoh, Yasuhiko Hayashi

**Affiliations:** 1 Graduate School of Natural Science and Technology, Okayama University, 3-1-1 Tsushima-naka, Kita-ku, Okayama, 700-8530 Japan; 2 Graduate School of Interdisciplinary Science and Engineering in Health Systems, Okayama University, 3-1-1 Tsushima-naka, Kita-ku, Okayama, 700-8530 Japan; 3 Graduate School of Environmental and Life Science, Okayama University, 1-1-1 Tsushima-naka, Kita-ku, Okayama, 700-8530 Japan

**Keywords:** Carbon Nanohorns, Cellulose, Skin sensitization, Composites, Bio-compatible

## Abstract

**Background:**

Conductive sheets of cellulose and carbon nanomaterials and its human skin applications are an interesting research aspect as they have potential for applications for skin compatibility. Hence it is needed to explore the effects and shed light on these applications.

**Method:**

To fabricate wearable, portable, flexible, lightweight, inexpensive, and biocompatible composite materials, carbon nanohorns (CNHs) and hydroxyethylcellulose (HEC) were used as precursors to prepare CNH-HEC (Cnh-cel) composite sheets. Cnh-cel sheets were prepared with different loading concentrations of CNHs (10, 20 50,100 mg) in 200 mg cellulose. To fabricate the bio-compatible sheets, a pristine composite of CNHs and HEC was prepared without any pretreatment of the materials.

**Results:**

The obtained sheets possess a conductivity of 1.83 × 10^− 10^ S/m and bio-compatible with human skin. Analysis for skin-compatibility was performed for Cnh-cel sheets by h-CLAT in vitro skin sensitization tests to evaluate the activation of THP-1 cells. It was found that THP-1 cells were not activated by Cnh-cel; hence Cnh-cel is a safe biomaterial for human skin. It was also found that the composite allowed only a maximum loading of 100 mg to retain the consistent geometry of free-standing sheets of < 100 μm thickness. Since CNHs have a unique arrangement of aggregates (dahlia structure), the composite is homogeneous, as verified by transmission electron microscopy (TEM) and, scanning electron microscopy (SEM), and other functional properties investigated by Raman spectroscopy, Fourier transform infrared spectroscopy (FT-IR), conductivity measurement, tensile strength measurement, and skin sensitization.

**Conclusion:**

It can be concluded that cellulose and CNHs sheets are conductive and compatible to human skin applications.

## Introduction

Among all the nanomaterials, carbon nanomaterials exhibit the most unique properties. Many prominent applications involving materials synthesized with carbon nanomaterials have gained popularity in recent years. Owing to their unique geometry, and properties closely related to single-walled carbon nanotubes (SWCNTs), carbon nanohorns (CNHs) have attracted significant attention. However, CNHs have different profiles of dispersion, structural geometry, surface chemistry, and synthesis methods and hence have a different property. The toxicity of carbon nanomaterials is also a significant limitation for bio-related applications. Many metals are used as catalysts for the synthesis of carbon nanotubes (CNT) and other carbon nanomaterials [1]. Owing to the inter-wall attraction force (van der Waals) of the CNTs, the dispersion of CNTs is a cumbersome process, and harsh chemical treatments using acids, and surfactants are necessary [2, 3].

CNHs may be an effective alternative to CNTs because they exhibit excellent purity and does not require post-synthesis treatment. CNHs have horn-shaped capped ends of approximately 2–3 nm, the wider ends aggregate to other CNHs, resulting in a Dahlia-like spherical structure [4]. The void between the tips of adjacent CNHs allows the CNH aggregates to have an active dispersion profile compared to CNTs. Although functionalization improved CNHs dispersion, the CNH aggregates could not be separated into single CNHs by chemical functionalization [5]. The three-dimensional arrangement of the CNHs aggregates allows electron transport and influences the bonding within the polymer along the surface, enhancing the mechanical properties of the composite, compared to the one-dimensional confinement of CNTs. Research on oxidized CNHs to entrap anticancer agents to treat lung cancer was reported as early as 2005 [6]. Polymer composites comprising poly-(vinyl alcohol), graphene, and CNHs have also been reported recently [7].

On the other hand, cellulose is an abundantly available natural polymer [8]. In particular, Hydroxyethylcellulose (HEC) is a derived cellulose that is used as a thickening agent, which are widely used materials in industries. HEC is also known for its application in the pharmaceutical industry for capsule formulations to improve hydrophilization of drugs [9]. Research on cellulose composites with carbon nanomaterials has been increasingly, with multiple reports on the numerous applications of cellulose-carbon nanomaterials. CNTs and cellulose composites have recently been studied as aerogels for vapor sensing [10] and, water sensors [11]. In addition, graphene oxide coated with cellulose nanofibers have been used for designing transparent conductive paper [12]. A few other have reports included the doping of conductive nanomaterials such as Ag nanowires, for electromagnetic interference shielding [13] and, PEDOT: PSS/MWCNT for supercapacitor electrodes [14].

In the area of smart [15], bio-compatible [16], wearable and implantable devices [17] carbon nanomaterials are being widely researched. To accommodate a device in close contact with human body/skin the compatibility of the material is a very crucial aspect. To evaluate allergic reactions and safety of different materials to human skin, will help in further exploring different applications of new materials. Such as, allergic dermatitis caused by skin sensitizers is one of the prominent items safety compliance considerations. To evaluate the skin sensitization potential of the prepared Cnh-cel sheets, h-CLAT, an in vitro skin sensitization test [including cytotoxicity test] was carried out. The cytotoxicity of CNH is expected to be low because of its spherical aggregates, as no metal catalyst is used during the synthesis of CNHs. CNHs and their composite materials are applicable to living bodies, especially in the medical field, safety evaluations, such as those evaluating toxicity and allergy, are strictly tested and regulated for safety.

For the safety evaluation of substances, allergic dermatitis is one of the essential items. After skin contact, substances causing allergic reactions, such as rash, are termed skin sensitizers, and the processes causing allergic reactions are termed skin sensitization (GHS 2017) [18]. The mechanisms of skin sensitization have been summarized in the form of an adverse outcome pathway (AOP) ranging from early events at the molecular level to adverse events through intermediate events, including the following four events (OECD 2014) [19]. The first event is the formation of covalent bonds between the electrophile; in other words, skin sensitizers covalently bind to the nucleophilic center of the proteins present in the skin. The second event includes inflammatory reactions, particularly in the keratinocytes in the skin, activation of the antioxidants/electrophilic substance responsive element (AREs) dependent signal transduction pathways. The third event is the activation of antigen-presenting cells called dendritic cells in the immune system, which is assessed by the expression of specific cell surface markers, chemokines, and cytokines. The fourth event is the proliferation of T cells, which play a central role in the host immune response. The evaluation of skin sensitization using animal (in vivo) and non-animal (in vitro) experiments aims to reproduce all or part of the AOP. In vivo animal experiments include guinea pig maximization (OECD 1992) [20] and the mouse regional lymph node test (OECD 2010) [21].

From the viewpoint of animal protection, regulations on animal experiments have become stricter in recent years. Particularly in the cosmetic industry in the EU, animal experiments for the production and evaluation of raw materials, processed products, and final products, have been banned (Ban on animal testing 2019). Additionally, sales and imports of such materials and products have also been banned; therefore, manufacturers exporting these to the EU are forced to comply. Thus, non-animal tests for the evaluation of skin sensitization are necessary. Currently, peptide binding tests (the first event of AOP) (OECD 2019 [22]; Gerberick et al. 2004, 2007) [23], keratinocyte reporter assay (the second event of AOP) [24–28], and human cell line activation test h-CLAT (the third event of AOP) are available and have been summarized in the OECD test guidelines as alternative evaluation methods (OECD, 2015a [29]; OECD, 2015b [28]; OECD, 2016 [30]).

The h-CLAT is a test method to evaluate the third event of the AOP by measuring the expression level of the cell surface marker (OECD, 2016) [31]. It is known that upon the activation of dendritic cells, the expression levels of cell surface markers such as CD86 and CD54 increase also [32]. In h-CLAT, the human monocytic leukemia cell line THP-1 cells were used as the dendritic cell model to evaluate the ability of test substances to activate dendritic cells.

In this study, we aimed to evaluate skin sensitization and cytotoxicity of novel composites of CNH and cellulose sheets using h-CLAT. These Cnh-cel sheets enabled the application of the novel composites in the field of restricted animal experiments.

### Experimental details

#### Cnh-cel sheet synthesis

**Fig. 1:**
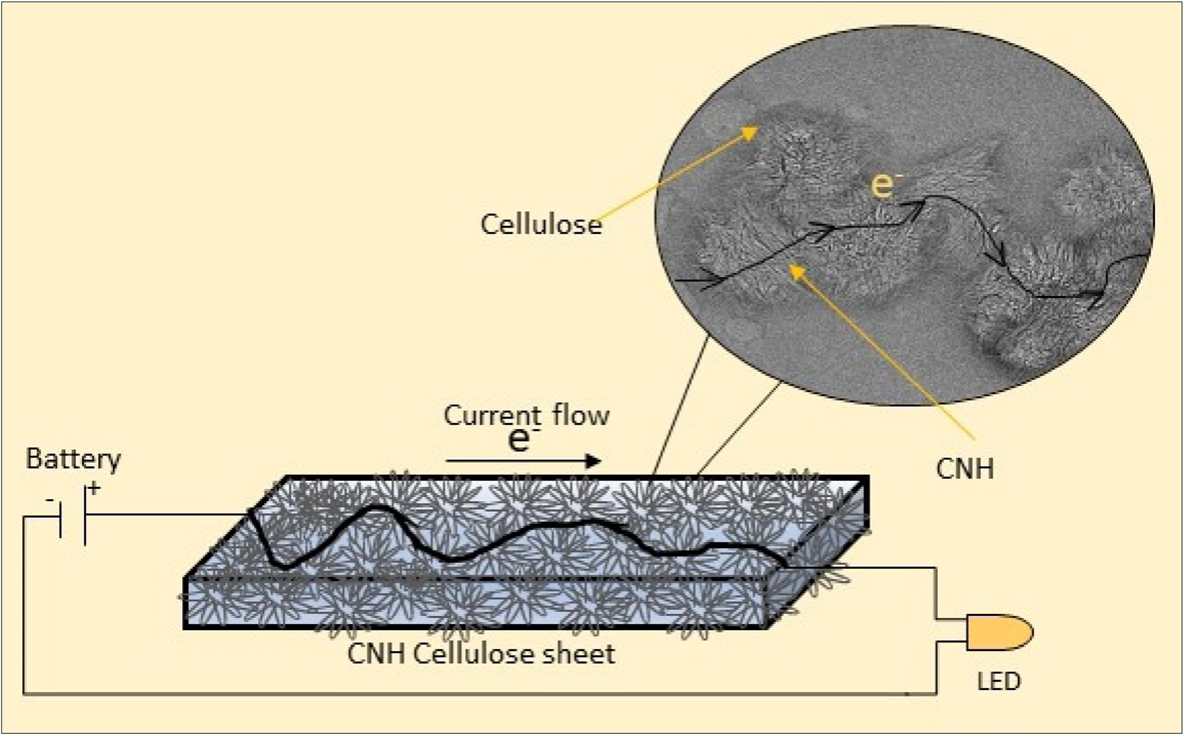
Graphical representation of a conductive path in the CNH-cellulose sheet

Hydroxyethylcellulose (HEC), carbon nanohorns (CNHs), and deionized (DI) water were used for cnh-cel sheet synthesis. A standard solution of HEC was prepared by dissolving 200 mg of cellulose in DI water. The cellulose nanofibers, when dissolved, can untangle or dissociate by subjecting them to ultra-sonication at a frequency of 23 kHz for 30 min. The cellulose solution was heated at 85 °C for 30 min to completely dissolve the cellulose fibers, which resulted in a transparent solution. CNHs were dispersed in DI water at loading concentrations of 10 mg, 20 mg, 50 mg, and 100 mg. The CNH solution was sonicated for 1 h. Initially, the CNHs precipitated due to their agglomeration and dahlia-like structures. When subjected to low-frequency ultra-sonication, an immediate dispersion was observed. The ratio of CNH to DI water was maintained at a very high rate, such that CNH exhibited enough room to disperse from their aggregated dahlia-like structures. Both solutions were divided into different portions depending on the ratios of HEC:CNH and then sonicated for 30 min to produce a volatile solution of different ratios of HEC:CNH which was agitated thoroughly. The combined solution was heated with stirring initially at 80 °C for 4 h at 200 rpm; to evaporate excess DI water, and the viscosity of the volatile solution started to increase. Subsequently, no excess DI water could be evaporated from the concentrated solution; the heat was raised to 90 °C for an hour and then finally to 100 °C. This resulted in a highly viscous, solution. The final solution was poured into a syringe, and a few droplets of this solution were dropped onto a clean glass plate to draw uniform sheets using the leveling applicator (bar coater). The sheets were allowed to dry at room temperature, and trapped a few molecules of water to maintain the structure. After a few minutes, the sheets were peeled off from the glass plate and analyzed using TEM, SEM, Raman, FT-IR, as well as tensile strength, and conductivity measurements, and the skin sensitization test. A schematic of the graphical representation of the Cnh-cel sheet, and its conductive path is shown in Fig. 1.

### Characterization

JEOL JEM-2100F High-resolution Transmission electron microscopy was used at an operating voltage of 200 kV to capture images at high magnifications. A JEOL JSM 6060 LA scanning electron microscope was used to observe the surface morphology of the Cnh-cel sheets and different magnifications. For RAMAN analysis, a JASCO NRS-5100 Nps laser Raman spectroscope was used to characterize the structural fingerprint of molecules present in the Cnh-cel sheets. To understand the functional groups present in the composite sheets, JASCO FT-IR 4100 was used. Tensile strength measurement was performed using a SHIMADZU AGS-X 5 N. Conductivity of Cnh-cel sheets was measured using ADCMT 6423 DC voltage current source/monitor.

## Results

### Transmission Electron microscopy

To investigate the interparticle interaction between the CNH aggregates, a transmission electron microscope was employed. Pristine carbon nanohorn samples were prepared by sonicating pristine samples in ethanol and DI water, and drop-casting on a copper grid.

**Fig. 2:**
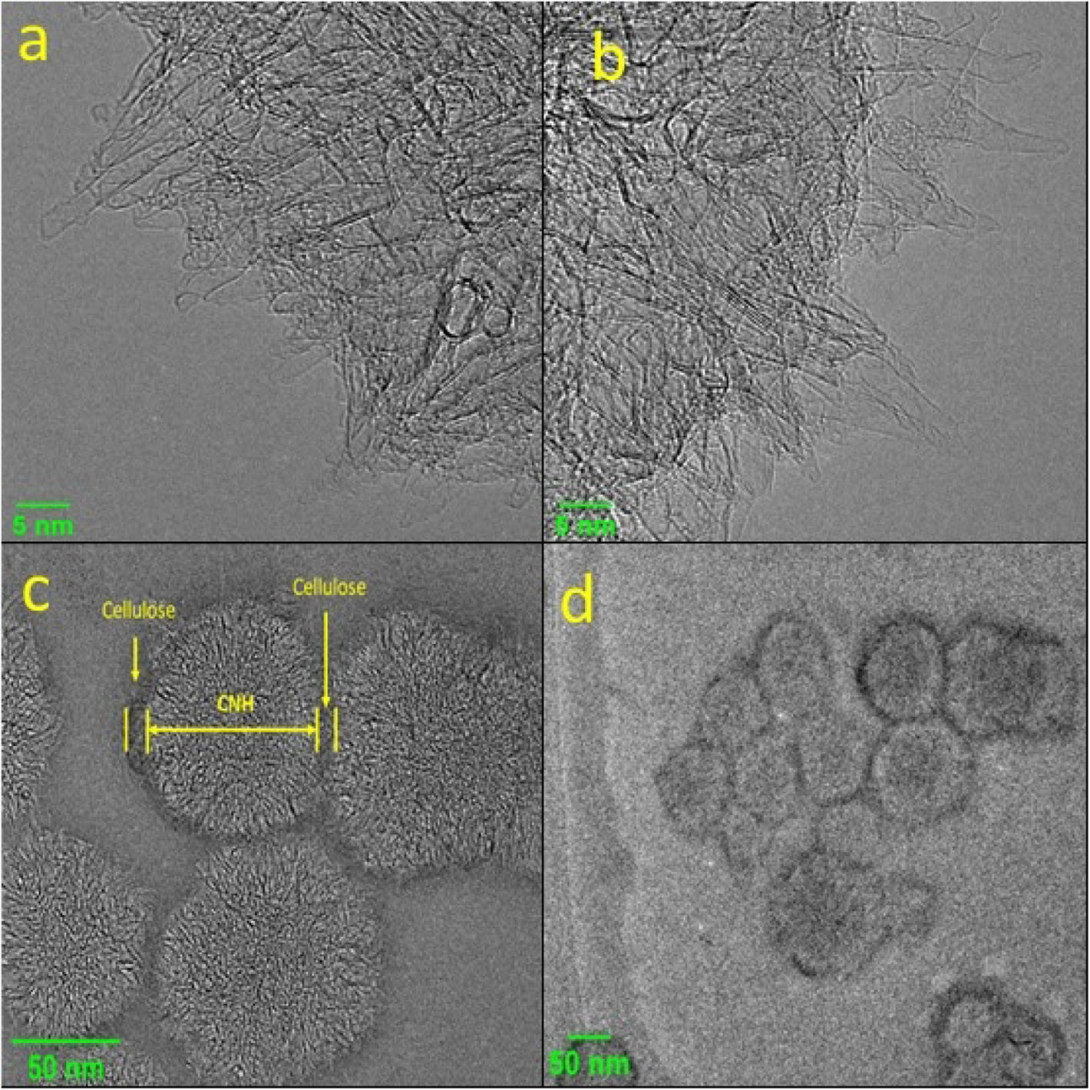
Pristine CNHs (**a**) and (**b**) observed under TEM at 5 nm magnification (**c**) and (**d**), showing the interaction of cellulose and CNHs at 50 nm magnification

Similarly, cellulose samples doped with CNHs were diluted with DI water and drop-cast on to the copper grid and dried on a hot plate for a few minutes. As shown in the TEM images in Fig. 2a and b, the pristine samples of CNH have cone-like structures, with single and double-walled CNHs arranged in dahlia-like structures, with the conical tip prodding out of the dahlia-like assembly. The pristine CNHs have cone tips of 2–3 nm in thickness. Figure 2c shows that these dahlia-like structures are accompanied by a layer of cellulose coating on the outer side connecting adjacent dahlia-like structured CNHs. This shows that the cellulose has adhered both in between and on top of the conical tips of the CNHs. As shown in Fig. 2d, a distorted ring-like structure of amorphous carbon surrounding the dahlia-like structures, can be seen when magnified at 50 nm using TEM.

### Scanning electron microscopy

**Fig. 3:**
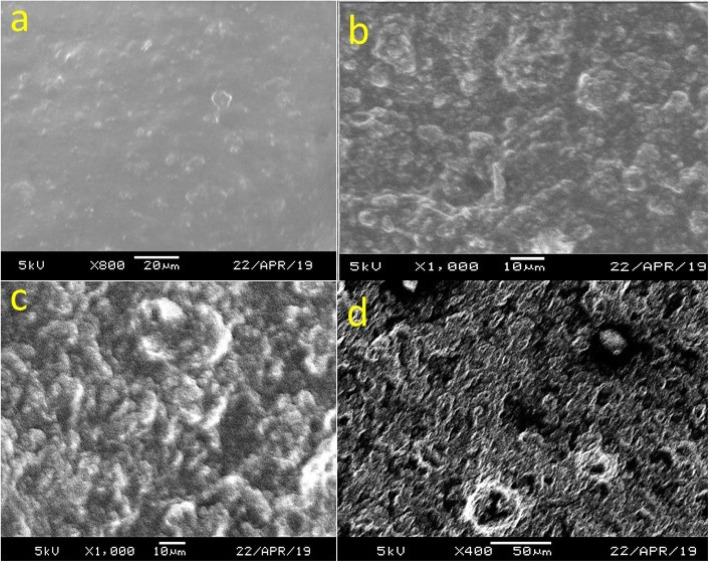
SEM images of the surface morphology of Cnh-cel sheets at different loading concentrations of CNH **a** 10 mg, **b** 20 mg, **c** 50 mg, **d** 100 mg

Scanning electron microscopy of the Cnh-cel sheets is shown in Fig. 3a – d. As the CNH loading concentration increase, the smoothness of the surface of the Cnh-cel sheets changes gradually. For the analysis of the cnh-cel sheets, a squared shape of the sheets is cut and used to observe under the scanning electron microscopy.

### Raman spectroscopy

**Fig. 4:**
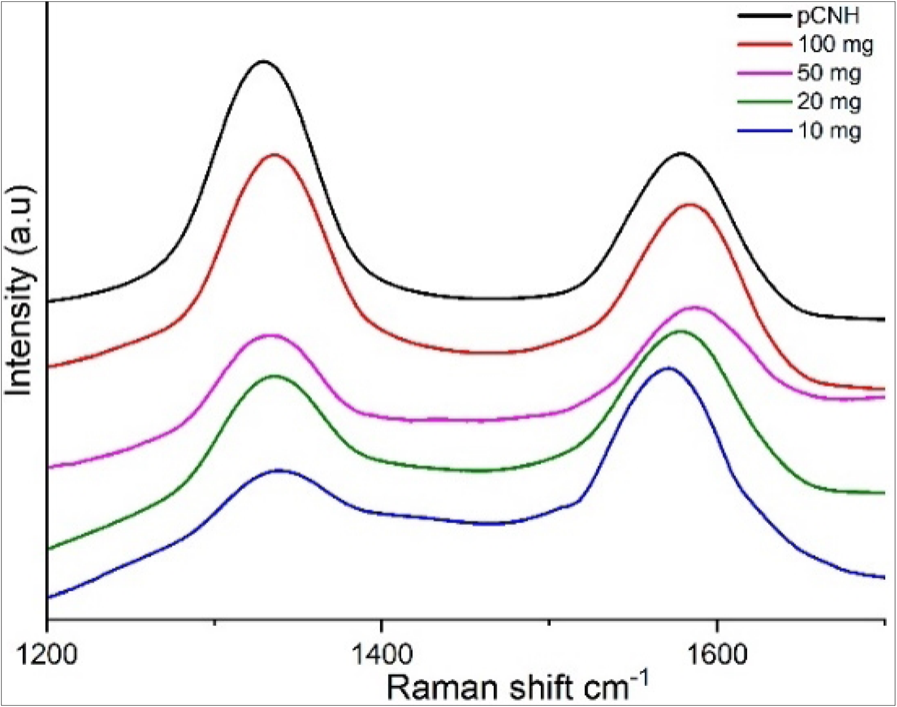
Raman plot showing I_d_ and I_g_ of CNHs at different loading concentrations

Raman spectroscopy characterization is a useful method of analysis to evaluate the purity of carbon nanomaterials. As interpreted from the plot in Fig. 4, CNHs exhibit two bands at 1338 cm^− 1^ (D band) and 1580 cm^− 1^ (G-band) [33].

### Fourier transform infrared spectroscopy

**Fig. 5:**
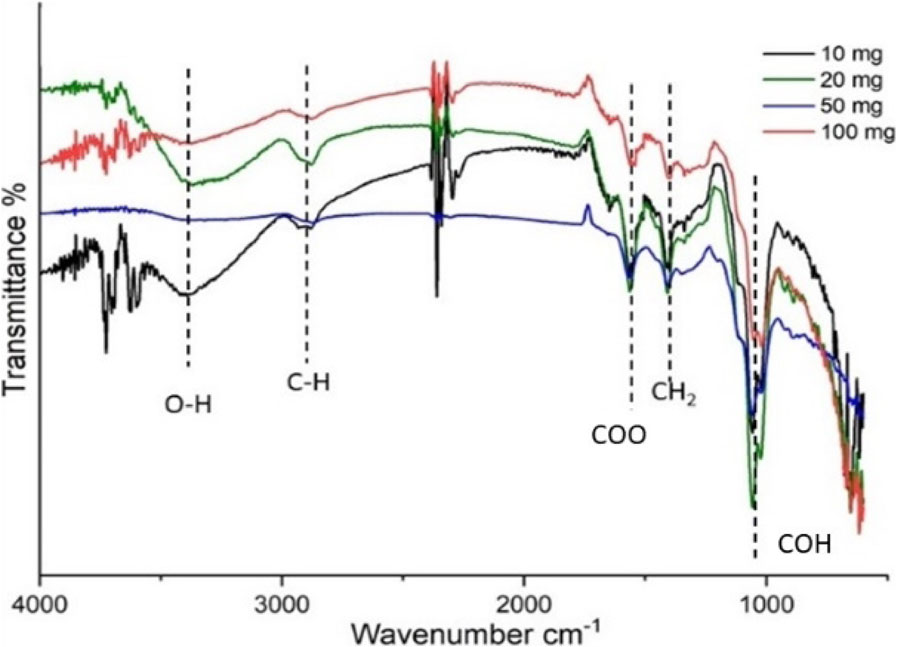
Showing FT-IR plot of different functional groups present in the Cnh-cel sheets

FTIR analysis of Cnh-cel sheets revealed the presence of a variety of functional groups in the Cnh-cel sheets. All the different loading concentrations of Cnh-cel sheets have a similar type of functional group fingerprint, as shown in Fig. 5.

### Tensile strength measurement

Tensile strength measurement is a crucial component in determining the durability and strength of the sheets. In a typical setup for the measurement of the tensile strength of a Cnh-cel sheets, a 20 × 10 mm sample of the sheets is cut and attached to the paper mold used for testing similar protocol mentioned elsewhere [34, 35]. The effective test area of the Cnh-cel sheets under tensile strength measurement is 10 × 10 mm.

### Conductivity measurement

A typical measurement of conductivity for sheets is determined by its area of cross-sectional area and length. However, the conductivity varies with the variation in the length and thickness of sheets. By keeping the parameters of length and thickness to a certain roundoff, the conductivity is determined as shown in Fig. 8.

## Discussion

In the TEM images the interaction between the long chains of cellulose nanofibers and the carbon nanohorns can be observed. Therefore, it can be concluded that even without any surface modification of the CNHs, interactions between CNHs and cellulose can be achieved. It is also observed that each dahlia-like aggregate of CNHs has a uniformly layered covering of cellulose; showing all the virtuous composites of cellulose and CNHs. This covering of cellulose to the CNH aggregates makes the CNHs aggregate as core-shell type nanoparticles, which can also be used in other applications where dissociation of the CNHs is not necessary and the dahlia-like structure is kept intact while allowing the CNHs to interact with other molecules via the cellulose shell. The interaction between the CNHs and cellulose can also be confirmed by FTIR analysis.

As per the SEM images the 100 mg doping concentration of CNH shown in Fig. 3d, the surface morphology of the Cnh-cel sheet is rough and porous compared to the 10 mg doping, as shown in Fig. 3a. In addition, as in Fig. 3b and c, the CNH doping concentrations are 20 mg and 50 mg, respectively, and the surface morphology is greatly varied. This indicates that the cellulose concentration in the Cnh-cel sheets is responsible for the surface smoothness and strength, as confirmed using the tensile strength measurement. In conclusion, the magnified images of the surface of the Cnh-cel sheets show that the strength and conductivity are inversely related to the concentration of the cellulose and CNHs.

In the Raman analysis the D band is observed because of the sp^3^ carbon, which produces elastic scattering. The intensities of I_d_ and I_g_ correspond to the sp^3^ and sp^2^ hybridization of carbon atoms present in the CNHs (I_d_/I_g_ = 1.2). When pristine CNH is compared to the other loading concentrations of CNH with cellulose the I_d_ band intensity is reduced gradually as the loading concentration is decreased. The elastic scattering of I_d_ band is affected significantly more that I_g_ band indicating the increase in amorphous carbon content in the cnh-cel sheets. Similarly it is a well-known fact that the cellulose is a hygroscopic material, hence few bonds are related to the hygroscopic property are expected in FT-IR analysis, and OH^−^ bonds are detected at 3340 cm^− 1^ also involving weak C-H stretching at 2896 cm^− 1^, both of which explain the hygroscopic nature of cellulose. Furthermore, an absorption band at 1065 cm^− 1^ corresponding to the ^−^(COH) functional group was also observed, and also a COO functional group is was identified at 1562 cm^− 1^. The intensities of different loading concentrations are represented by the intensity peaks in Fig. 5. As the doping concentration of CNH increases from 10 mg to 100 mg the intensity of ^−^(COH) functional group is decreased. Also, OH^−^ peak is affected by the increase in the doping of CNH which could explain the tensile strength decreasing of the cnh-cel sheets by increasing the loading concentration.

**Fig. 6:**
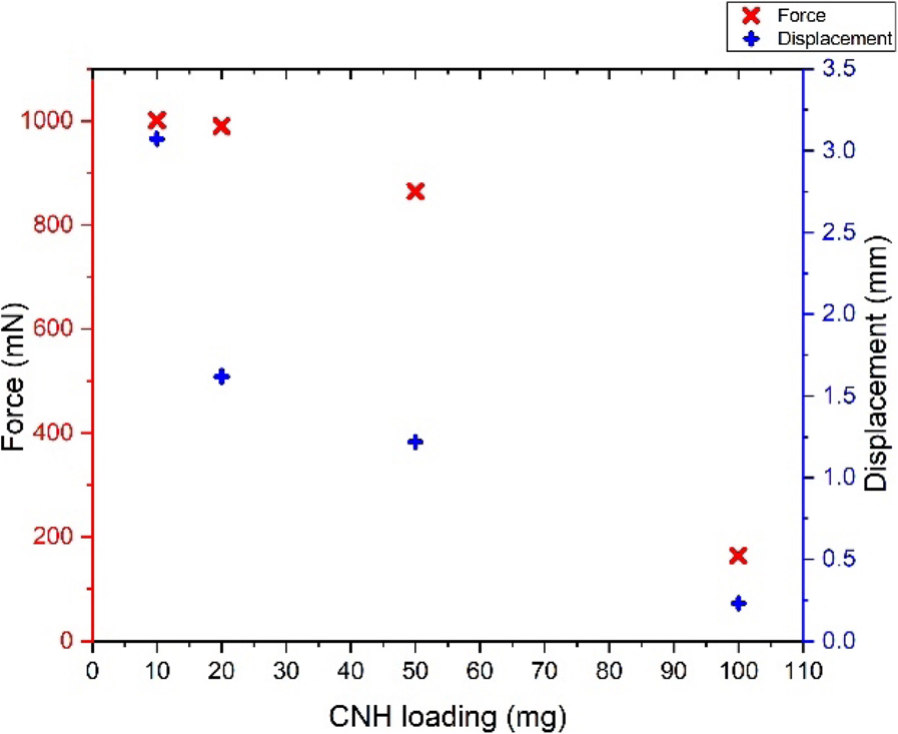
Tensile strength plot of Force vs Displacement vs CNH loading concentrations

**Fig. 7:**
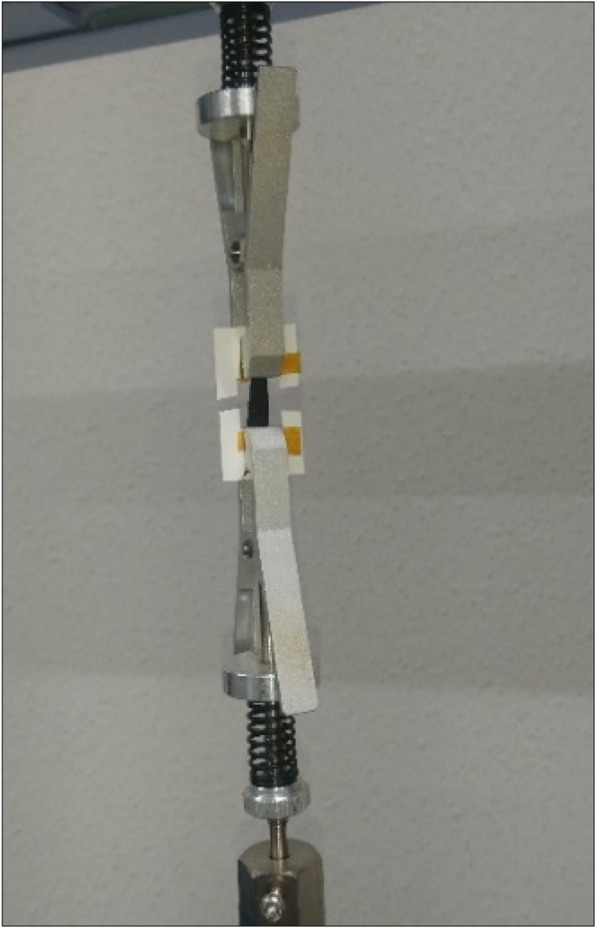
Showing Cnh-cel sheet tensile measurement setup

In the Tensile measurement it is observed that, as the CNH loading increases, the strength decreases as the CNHs loading reduces the interparticle bonding between the adjacent cellulose molecules. The CNH loading concentration of 10 mg had a displacement of 3.074 mm having an ultimate strength to withstand a force of 1001.50 mN, as shown in Fig. 6. In contrast, the 20 mg loading was able to withstand a displacement of 1.62 mm but had an ultimate strength to tolerate a force of 990.7 mN until they broke. This shows that when the loading concentration is doubled, and the displacement was reduced by 52.7%, but the ultimate strength was only affected by 9.8%. Similarly, at 50 mg loading, the displacement was 1.2 mm at which it could stand 865.79 mN of force, whereas for 100 mg loading the displacement was 0.23 mm for the ultimate strength to bear the force of 164.24 mN. The threshold of the force endurable for Cnh-cel sheets for pristine Cnh-cel composite are collectively plotted in CNH loading versus force, and the displacement is shown below in Fig. 6. The plot shows a trend of a decrease by the force and displacement proportional to the loading concentration, which enables us to understand the suitable loading concentrations based on the type of application. It was found that a further increase in the loading concentration of CNHs could not provide structural integrity to fabricate thin films of less than 100 μm in thickness, which could be related to the upper limit of doping, possibly for pristine CNHs to cellulose ratios. However, functionalization of the CNHs and dissolving the cellulose in ionic liquids [36] could increase the endurance of Cnh-cel composite sheets, while compromising other properties. A typical setup of the tensile strength measurement of a Cnh-cel sheet is shown in Fig. 7.

**Fig. 8:**
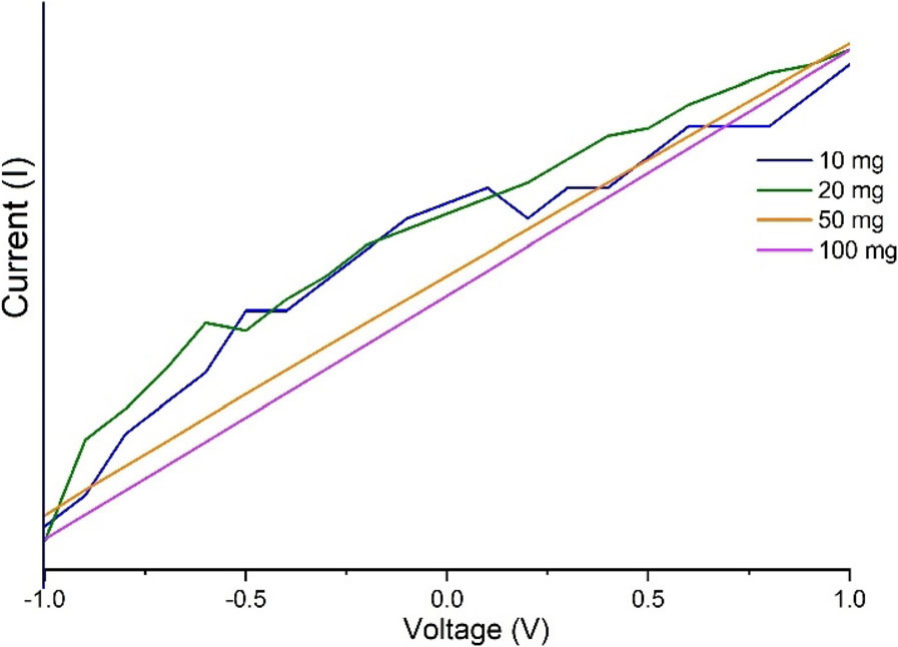
V-I curve of Cnh-cel sheets at different loading concentrations from − 1 V to + 1 V

The conductivity measurements are proportional to the level of doping of CNH in the cellulose matrix as the CNH doping increases the conductivity of the sample by 100%. However, we could not determine the maximum doping possible to retain the geometry of the thin film for < 100 μm sized sheets at 50 wt% cellulose. Figure 8 shows the combined plot of the voltage (V) vs. current (I), however, the current values are different for all the Cnh-cel sheets. The conductivity of the sheets with 10 mg and 20 mg loading of CNHs show a non-linear V-I curve, which indicates that the sheets are not good conductors, and the conductivity cannot be determined as the sheets do not have a linear V-I curve. However, at 50 and 100 mg loading of CNHs, a linear V-I curve is observed, which translates to a conductivity of 6.145 × 10^− 11^ S/m for 50 mg loading and 1.83 × 10^− 10^ S/m for 100 mg loading.

### The skin sensitization test method is outlined as follows

This study was conducted according to the OECD Guidelines for the Testing of Chemicals (OECD, 2016).

### Cell culture

THP-1 cells (RCB1189, Riken, RBC, Tsukuba, Japan) were cultured in an incubator (37 °C and 5% CO_2_) using RPMI-1640 medium (Nacalai Tesuque, Kyoto, Japan) supplemented with 10% fetal bovine serum (FBS) (Nichirei Biosciences, Tokyo, Japan), 0.05 mM 2-mercaptoethanol, 100 units/mL penicillin in 100 μg/mL streptomycin (both Nacalai Tesuque). The cells were seeded at a density of 0.1–0.2 × 10^6^ cells/ mL once every 2 to 3 days to maintain a density of 0.1–1.0 × 10^6^ cells/mL.

### Determination of test dose

Three doses of working solutions were prepared as follows. Cnh-cel composites were added to the cell culture medium at a final concentration of 8666 μg/mL. This working solution was serially diluted at 1:2 to prepare the remaining two working solutions. Eighty microliters of the cell suspension at a density of 2 × 10^6^ cells/mL was seeded into each well of a 96-well plate, and the cells were added with 80 μL working solutions or the medium (negative control) and cultured in an incubator for 24 h. Cells were then collected into 1.5 mL tubes and centrifuged at 250×g, 4 °C, for 5 min. Cells were resuspended in 500 μL of FACS buffer (PBS supplemented with 0.1% BSA). After 10 washes with repetitive resuspension and centrifugation, the cells were re-suspended in 200 μL FACS buffer. Immediately before measurement, the cells were stained by adding 5 μL propidium iodide (PI) (Invitrogen, Carlsbad, CA) solution (20 μg/mL diluted with FACS buffer). Cell viability was measured using flow cytometry, and the concentration (CV75) of the test sample in 75% cell viability was determined using Formula 1.

$$ \mathrm{Formula}\ 1\ \log CV75=\frac{\left(75-\boldsymbol{c}\right)\times \mathit{\log}\left(\boldsymbol{b}\right)-\left(75-a\right)\times \mathit{\log}\left(\boldsymbol{d}\right)}{\boldsymbol{a}-\boldsymbol{c}} $$

**a** is the minimum value of cell viability over 75%

**c** is the maximum value of cell viability below 75%

**b** and **d** are the concentrations showing the value of cell viability **a** and **c,** respectively

### Skin sensitization test

Four doses of Cnh-cel composite working solutions at concentrations of CV75 × 1.2^–2, − 1, 0, 1^ were prepared. Cells were seeded, working solutions added, cultured, and washed as described above. After centrifugation, the cells were blocked with 330 μL of blocking solution (FACS buffer containing 0.01% human globulin Cohn fraction II, III (Sigma-Aldrich, St. Louis, MO)) for 15 min at 4 °C. The cells were divided into three tubes with 100 μl and added 30 μl of diluted anti-CD86 (BD-PharMingen, Franklin Lakes, NJ), anti-CD54 or mouse IgG 1 (both Dako, Santa Clara, CA) antibodies. To make the diluted antibody solutions, the antibodies and FACS buffer were mixed at the ratios between antibody solutions and FACS buffer were at 6:44, 3:47, and 3:47, respectively. After 30 min at 4 °C, cells were washed with FACS buffer twice and re-suspended in 150 μL of FACS buffer followed by the addition of 4 μL of the diluted PI solution. The relative fluorescence intensity (RFI) was calculated using Formula 2 based on the measured mean fluorescence intensity (MFI) obtained by flow cytometry, as an indicator of CD86 and CD54 relative expression.

$$ \mathrm{Formula}\ 2\  RFI=\frac{\begin{array}{c} MFI\  of\ chemical\ treated\ cells-\\ {} MFI\  of\ chemical\ treated\ isotype\ control\ cell\end{array}}{\begin{array}{c}\mathrm{MFI}\ \mathrm{of}\ \mathrm{vehicle}\ \mathrm{control}\ \mathrm{cells}-\\ {}\mathrm{MFI}\ \mathrm{of}\ \mathrm{vehicle}\ \mathrm{isotype}\ \mathrm{control}\ \mathrm{cells}\end{array}}\times 100 $$

### In vitro skin sensitization test

**Fig. 9:**
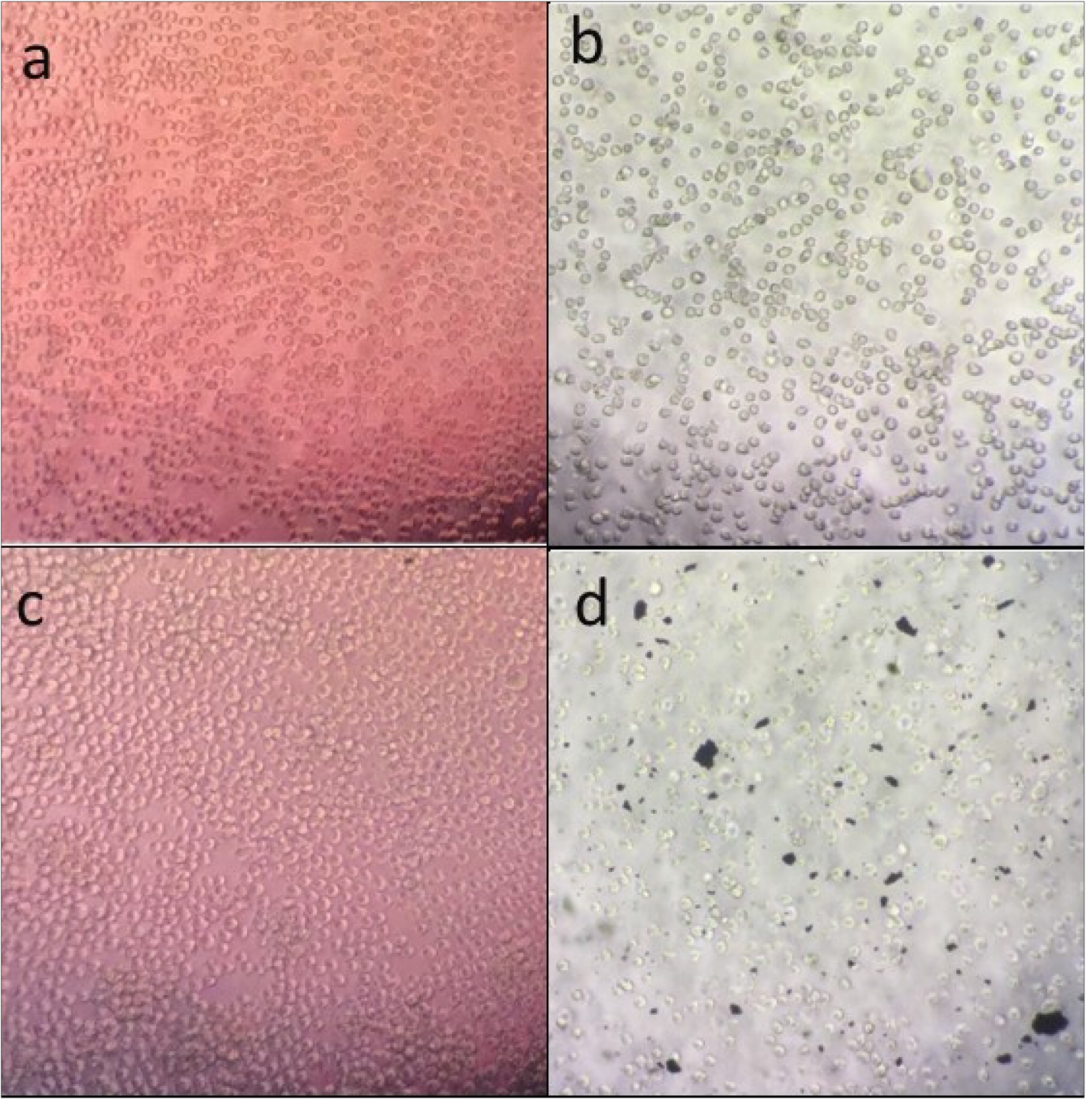
A typical example of the cells used in this study

To evaluate the Cnh-cel sheets in skin sensitization test, the CV75, the does whose cell viability is 75%, defined in Formula 1 was determined as 1999 μg/mL using experimentally obtained parameters, a = 89.5%, b = 1083 μ/mL, c = 73.1%, d = 2166 μg/mL. The test doses, CV75 * 1.2^(1, 0, − 1, and − 2) were then calculated as 2399 μg/mL (CV75 × 1.2), 1999 μg/mL (CV75), 1666 μg/mL (CV75/1.2), 1388 μg/mL (CV75/1.22), respectively. The results of the cytotoxicity and test dose determination tests are shown in Fig. 9. The CV 75 value was determined using Formula 1.

In this test, **a** = 89.5%, **b** = 1083 μ/mL, **c** = 73.1%, **d** = 2166 μg/mL. The CV75 value of Cnh-cel composites was 1999 μg/mL. The exposure concentration of the Cnh-cel composites was set at four levels, diluted at a ratio of 1–2 based on CV75. The value were 2399 μg/mL (CV75 × 1.2), 1999 μg/mL (CV75), 1666 μg/mL (CV75/1.2), 1388 μg/mL (CV75/1.2^2^).

**Fig. 10:**
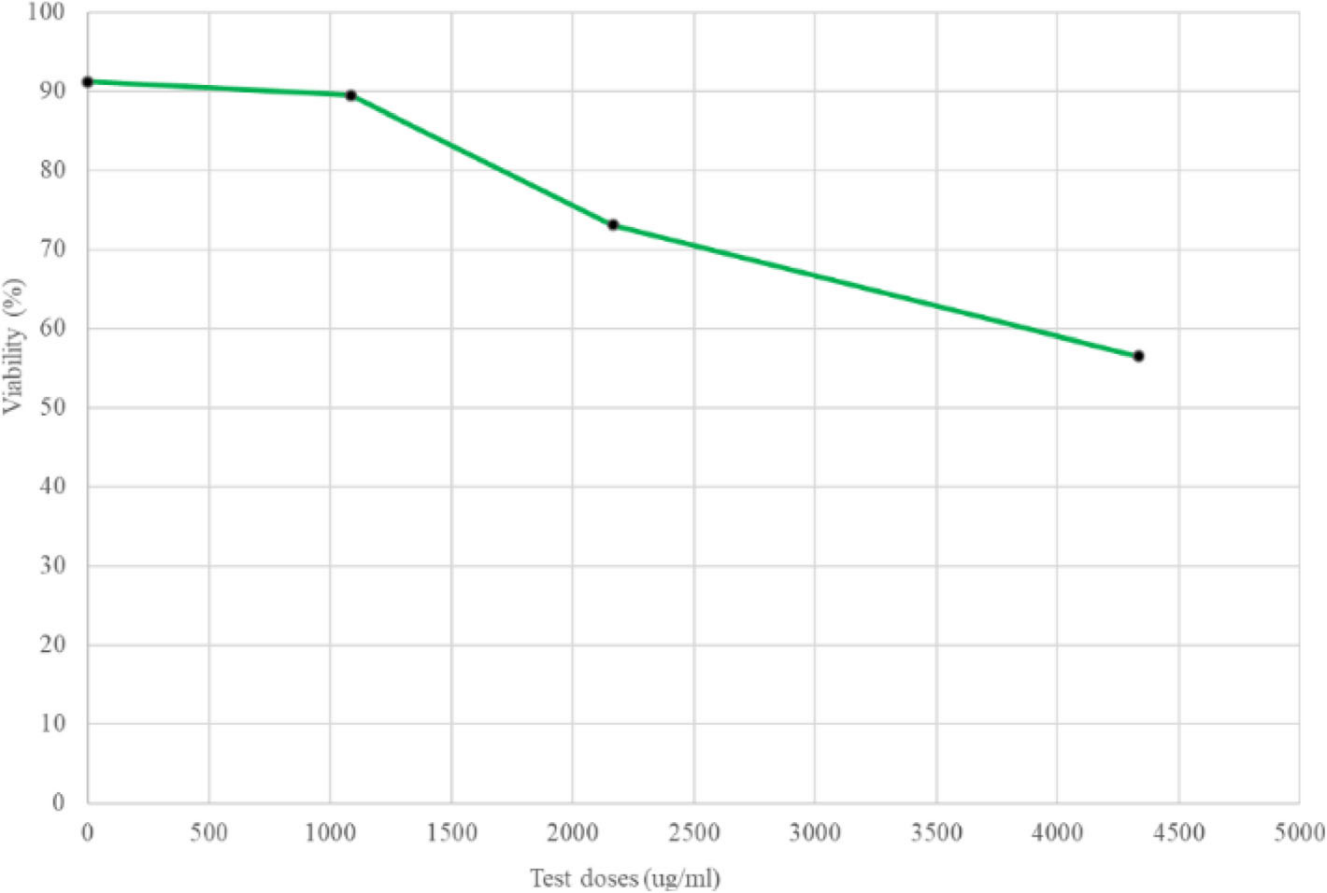
Cell viability effects of three test doses

Cell viability was also confirmed microscopically shown in Fig. 9. Note that the Cnh-cel composites appeared to be a little toxic at the higher concentrations shown in Fig. 10. However, the CV75 of Cnh-cel composites was much higher than that of lactic acid, which is a standard negative control in this test (1000 μg/mL) suggesting that the cytotoxicity of the Cnh-cell composites is very low. The concentration of Cnh-cel composites and cell viability were inversely proportional (Fig. 10). Thus, a high concentration of Cnh-cel composites is cytotoxic. The CV75 value of the Cnh-cel composite was 1999 μg/mg. The CV75 value of lactic acid, which is used in cosmetics suggests a relatively low cytotoxicity value of 1000 μg/mL. Given the comparison of the CV75 values, the cytotoxicity of the Cnh-cel composites is relatively low.

Aliquots of cells were taken from the assays and imaged (a) just before starting the assay, (b) after washing cells and centrifugation, (c) 24 h after cells incubated with negative control, (d) 24 h after cells incubated with Cnh-cell composites and centrifuged. Media control (b) Media control (incubate 24 h) (c) Media control (after centrifugation) (d) Cnh-Cell sheets (after centrifugation).

**Fig. 11:**
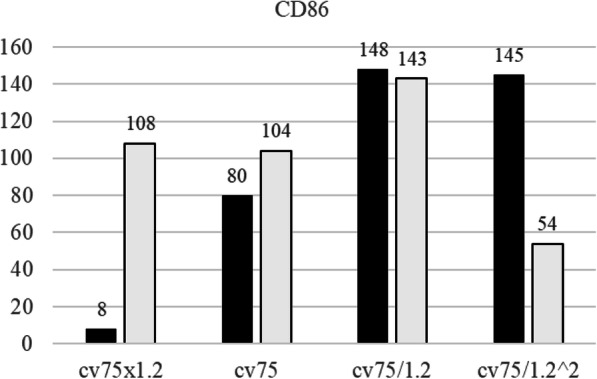
Relative florescence intensity of CD86

**Fig. 12:**
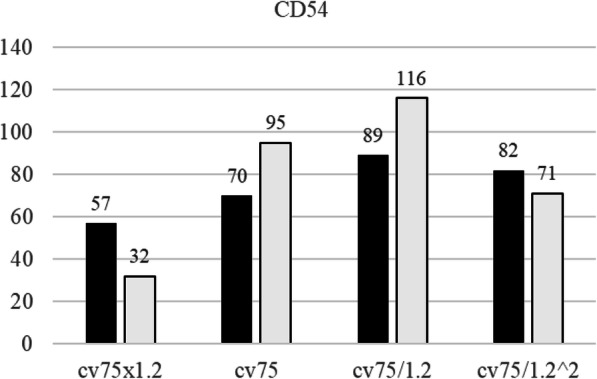
Relative florescence intensity of CD54

The skin sensitization is defined as the RFI (the relative fluorescence intensity calculated by Formula 2) of CD86 ≥ 150 and CD54 ≥ 200. As shown in Figs. 11 and 12, the RFIs of CD86 and CD54 were lower than 150 and 200, respectively, in all the tested cases indicating that the Cnh-cell composites are not skin sensitization agents. Note the assay was performed twice. Each is shown in the closed bars and open bars in Supplementary Figure S1 and Supplementary Figure S2. The results of the skin sensitization tests are shown in Figs. 11 and 12. The results demonstrate that the RFI values of CD86 and CD54 are lower than the positive criteria (CD86 RFI ≥ 150, CD54 RFI ≥ 200). Therefore, Cnh-cel composites were identified as skin sensitization-negative in this experiment.

THP-1 cells were incubated with Cnh-cel composites (three doses: 4333 μg/mL, 2166 μg/mL, 1083 μg/mL) or medium (negative control, 0 μg/mL) for 24 h. The cells were stained with PI solution. Cell viability was measured using flow cytometry.

**Fig. 13:**
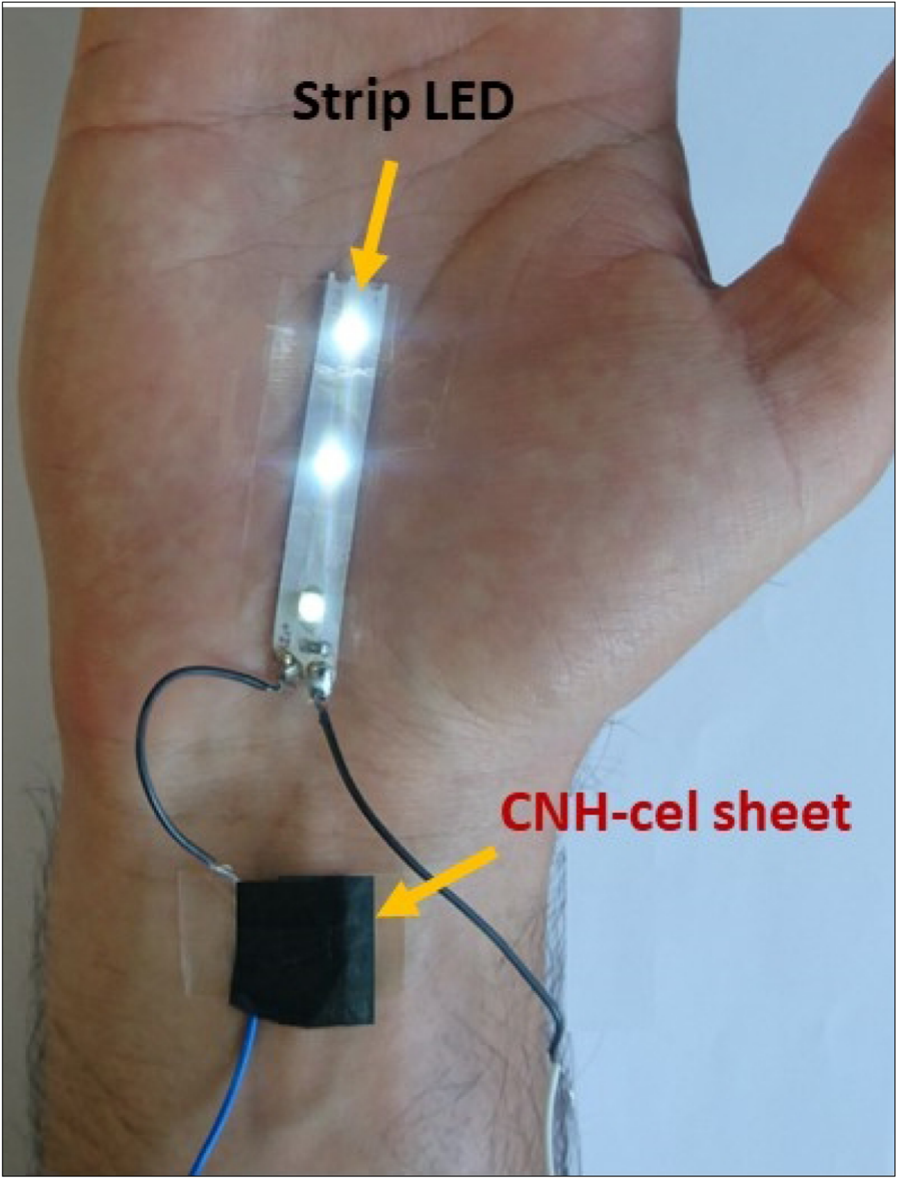
The application of Cnh-cel sheets on human skin and passage an electric current to light up LEDs

THP-1 cells were incubated with Cnh-cel composites (four doses) for 24 h. The cells were stained with anti-CD86, anti-CD54, or mouse IgG-1 antibodies. MFI was measured using flow cytometry. RFI was calculated using MFI. The black bar represents the first result, and the white bar represents the second result. The Cnh-cel composites treated group showed that RFI values of CD86 and CD54 are lower than the positive criteria (CD86 RFI ≥ 150 and CD54 RFI ≥ 200). A working example of Cnh-cel sheets attached to human skin with glowing LED lights is shown in Fig. 13. Notably, these Cnh-cel sheets did not exhibit skin sensitization in other bioassays, such as OECD TG442C (Amino acid Derivative Reactivity Assay) or OECD TG442D (data included in Supplementary Information).

## Conclusion

Cellulose carbon nanohorn composite sheets (Cnh-cel sheets) with different loading concentrations were successfully fabricated in our study. The properties of the Cnh-cel sheets were analyzed and determined using various characterization techniques. The conductivity of the Cnh-cel sheet at the maximum loading concentration was measured to be 1.88×10^-10^ S/m, and the skin application of the Cnh-cel sheets to human skin was also tested, and it was proven to be safe for applications on human skin. These initial experiments of Cnh-cel sheets shed light on the understanding of carbon allotropes application as smart materials. The encouraging properties of Cnh-cel sheets such as flexibility, strength, conductive, and skin-compatible indicate their high potential for various modern-day applications.

## Supplementary information

**Additional file 1.**

## Data Availability

All data generated and analyzed during the current study are available with the corresponding author upon reasonable request.

## References

[1] Hoyos-Palacio LM, García AG, Pérez-Robles JF, González J, Martínez-Tejada HV. Catalytic effect of Fe, Ni, Co and Mo on the CNTs production. InIOP conference series: materials science and engineering 2014 (Vol. 59, No. 1, p. 012005). IOP Publishing.

[2] Osorio AG, Silveira ICL, Bueno VL, Bergmann CP (2008). H2SO4/HNO3/HCl-Functionalization and its effect on dispersion of carbon nanotubes in aqueous media. Appl Surf Sci.

[3] Vaisman L, Wagner HD, Marom G (2006). The role of surfactants in dispersion of carbon nanotubes. Adv Colloid Interf Sci.

[4] Iijima S, Yudasaka M, Yamada R, Bandow S, Suenaga K, Kokai F, Takahashi K. Nano-aggregates of single-walled graphitic carbon nano-horns. Chem Phys Lett. 1999;309(3-4):165-70.

[5] Cioffi C, Campidelli S, Sooambar C, Marcaccio M, Marcolongo G, Meneghetti M (2007). Synthesis, characterization, and photoinduced electron transfer in functionalized single wall carbon nanohorns. J Am Chem Soc.

[6] Ajima K, Yudasaka M, Murakami T, Maigné A, Shiba K, Iijima S (2005). Carbon nanohorns as anticancer drug carriers. Mol Pharm.

[7] Kadambi SB, Pramoda K, Ramamurty U, Rao CNR (2015). Carbon-Nanohorn-reinforced polymer matrix composites: synergetic benefits in mechanical properties. ACS Appl Mater Interfaces.

[8] Kroschwitz JI, Seidel A. Kirk-Othmer encyclopedia of chemical technology. 2004.

[9] Lerk CF, Lagas M, Fell JT, Nauta P (1978). Effect of hydrophilization of hydrophobic drugs on release rate from capsules. J Pharm Sci.

[10] Qi H, Liu J, Pionteck J, Pötschke P, Mäder E (2015). Carbon nanotube-cellulose composite aerogels for vapour sensing. Sensors Actuators B Chem.

[11] Qi H, Mäder E, Liu J (2013). Unique water sensors based on carbon nanotube-cellulose composites. Sensors Actuators B Chem.

[12] Gao K, Shao Z, Wu X, Wang X, Li J, Zhang Y (2013). Cellulose nanofibers/reduced graphene oxide flexible transparent conductive paper. Carbohydr Polym.

[13] Choi HY, Lee TW, Lee SE, Lim JD, Jeong YG (2017). Silver nanowire/carbon nanotube/cellulose hybrid papers for electrically conductive and electromagnetic interference shielding elements. Compos Sci Technol.

[14] Zhao D, Zhang Q, Chen W, Yi X, Liu S, Wang Q (2017). Highly flexible and conductive cellulose-mediated PEDOT:PSS/MWCNT composite films for supercapacitor electrodes. ACS Appl Mater Interfaces.

[15] Jang Y, Kim SM, Spinks GM, Kim SJ (2020). Carbon nanotube yarn for fiber-shaped electrical sensors, actuators, and energy storage for smart systems. Adv Mater.

[16] Aoki K, Saito N (2020). Biocompatibility and carcinogenicity of carbon nanotubes as biomaterials. Nanomaterials..

[17] Huang X, Wang L, Wang H, Zhang B, Wang X, Stening RYZ (2020). Materials strategies and device architectures of emerging power supply devices for implantable bioelectronics. Small..

[18] GHS (Rev.7) (2017) - Transport - UNECE. https://www.unece.org/trans/danger/publi/ghs/ghs_rev07/07files_e0.html.

[19] OECD. The Adverse Outcome Pathway for Skin Sensitisation Initiated by Covalent Binding to Proteins, OECD Series on Testing and Assessment. Paris: OECD Publishing; 2014. No. 168, p 105. 10.1787/9789264221444-en.

[20] OECD. Test No. 406: Skin Sensitisation, OECD Guidelines for the Testing of Chemicals, Section 4. Paris: OECD Publishing; 2019. 10.1787/9789264070660-en.

[21] OECD. Test No. 429: Skin Sensitisation: Local Lymph Node Assay, OECD Guidelines for the Testing of Chemicals, Section 4. Paris: OECD Publishing; 2010 p. 20. 10.1787/9789264071100-en.

[22] Gerberick GF, Vassallo JD, Bailey RE, Chaney JG, Morrall SW, Lepoittevin J-P (2004). Development of a peptide reactivity assay for screening contact allergens. Toxicol Sci.

[23] Gerberick GF, Vassallo JD, Foertsch LM, Price BB, Chaney JG, Lepoittevin J-P. Quantification of chemical peptide reactivity for screening contact allergens: a classification tree model approach. Toxicol Sci. 2007;97(2):417–27. 10.1093/toxsci/kfm064 17400584

[24] Emter R, Ellis G, Natsch A. Performance of a novel keratinocyte-based reporter cell line to screen skin sensitizers in vitro. Toxicol Appl Pharmacol. 2010;245(3):281-90. 10.1016/j.taap.2010.03.009 20307559

[25] Natsch A. The Nrf2-Keap1-ARE toxicity pathway as a cellular sensor for skin sensitizers-functional relevance and a hypothesis on innate reactions to skin sensitizers. Toxicol Sci. 2010;113(2):284-92. 10.1093/toxsci/kfp228. 10.1093/toxsci/kfp228 19767620

[26] OECD. Test No. 442D: In Vitro Skin Sensitisation: ARE-Nrf2 Luciferase Test Method, OECD Guidelines for the Testing of Chemicals, Section 4. Paris: OECD Publishing; 2018. 10.1787/9789264229822-en. Test method 20.

[27] TG 495: Ros (Reactive Oxygen Species) Assay for Photoreactivity. OECD; 2019. (OECD Guidelines for the Testing of Chemicals, Section 4). https://www.oecd-ilibrary.org/environment/tg-495-ros-reactive-oxygen-species-assay-for-photoreactivity_915e00ac-en.

[28] Test No. 442D: In Vitro Skin Sensitisation. OECD; 2018. (OECD Guidelines for the Testing of Chemicals, Section 4). https://www.oecd-ilibrary.org/environment/test-no-442d-in-vitro-skin-sensitisation_9789264229822-en.

[29] Test No. 442C: In Chemico Skin Sensitisation. OECD; 2019. (OECD Guidelines for the Testing of Chemicals, Section 4). https://www.oecd-ilibrary.org/environment/test-no-442c-in-chemico-skin-sensitisation_9789264229709-en.

[30] OECD. Test No. 442E: In Vitro Skin Sensitisation: In Vitro Skin Sensitisation assays addressing the Key Event on activation of dendritic cells on the Adverse Outcome Pathway for Skin Sensitisation, OECD Guidelines for the Testing of Chemicals, Section 4, p.21. Paris: OECD Publishing; 2018. 10.1787/9789264264359-en.

[31] Ashikaga T, Yoshida Y, Hirota M, Yoneyama K, Itagaki H, Sakaguchi H (2006). Development of an in vitro skin sensitization test using human cell lines: the human cell line activation test (h-CLAT). Toxicol Vitr.

[32] Aiba S, Terunuma A, Manome H, Tagami H (1997). Dendritic cells differently respond to haptens and irritants by their production of cytokines and expression of co-stimulatory molecules. Eur J Immunol.

[33] Lin Z, Iijima T, Karthik PS, Yoshida M, Hada M, Nishikawa T (2017). Surface modification of carbon nanohorns by helium plasma and ozone treatments. Jpn J Appl Phys.

[34] Kim H-I, Wang M, Lee SK, Kang J, Nam J-D, Ci L (2017). Tensile properties of millimeter-long multi-walled carbon nanotubes. Sci Rep.

[35] Liang B, Zhao H, Zhang Q, Fan Y, Yue Y, Yin P. Ca 2+ Enhanced Nacre-Inspired Montmorillonite−Alginate Film with Superior Mechanical, Transparent, Fire Retardancy, and Shape Memory Properties. ACS Appl Mater Interfaces. 2016;8(42):28816–23. 10.1021/acsami.6b08203 27726325

[36] Zhao D, Fu L, Zhang J, Li H, Liu M, Fu J (2012). Study on the dissolution of cellulose in <I>N</I>-Allylpyridinium chloride ionic liquid and co-solvent composites. Acta Polym Sin.

