# Supplementary material for: Synthesis and characterization of conductive flexible cellulose carbon nanohorn sheets for human tissue applications
Source: Biomater Res. 2020 Oct 23;24:18. doi: 10.1186/s40824-020-00194-3 (PMC7583293; doi:10.1186/s40824-020-00194-3)
Supplement: Supplementary file 1 — Additional file 1. [file 40824_2020_194_MOESM1_ESM.docx]

**Supplementary Information**

**Synthesis and characterization of conductive flexible cellulose carbon nanohorn sheets for human tissue applications**

Karthik Paneer Selvam^1^, Taichi Nagahata^2^, Kosuke Kato^2^, Mayuko Koreishi^2^, Toshiyuki Nakamura^3^, Yoshimasa Nakamura^3^, Takeshi Nishikawa^1^, Ayano Satoh^2*^, Yasuhiko Hayashi^1*^

^1^Graduate School of Natural Science and Technology, Okayama University, 3-1-1 Tsushima-naka, Kita-ku, Okayama, 700-8530 Japan

^2^Graduate School of Interdisciplinary Science and Engineering in Health Systems, Okayama University, 3-1-1 Tsushima-naka, Kita-ku, Okayama, 700-8530 Japan

^3^Graduate School of Environmental and Life Science, Okayama University, 1-1-1 Tsushima-naka, Kita-ku, Okayama, 700-8530 Japan

* hayashi.yasuhiko@okayama-u.ac.jp; Tel: +81-086-251-8230


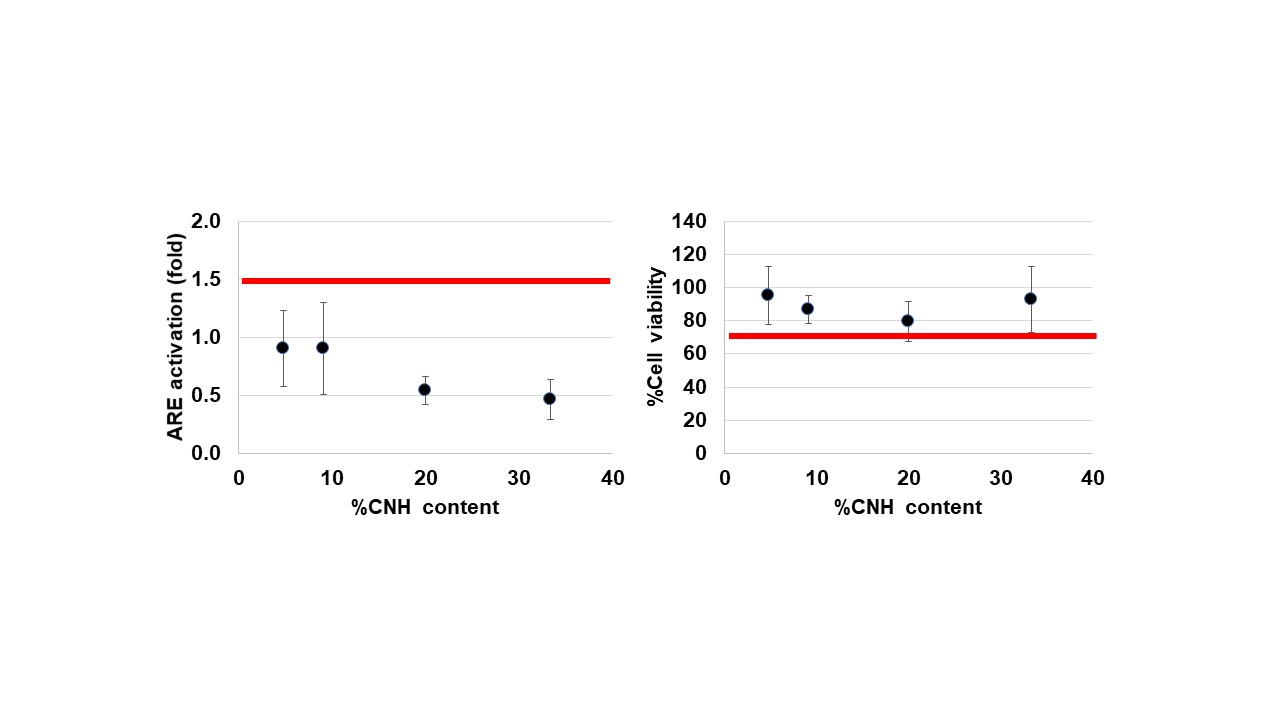


**Supplementary Figure S1**

The Cnh-cel sheets did not activate or kill keratinocytes according to the bioassay evaluating skin sensitization AOP#2, OECD TG442D (1.5> , %70< are the positive prediction lines, shown in red lines, respectively). Bars, SD (n=3). Note that no %CNH content dependency was detected (P>0.05, by ANOVA, https://goodcalculators.com/one-way-anova-calculator/).

Supplementary methods:

Human keratinocyte cell line, HaCaT was obtained from German Cancer Research Center (DKFZ, Heidelberg, Germany) and maintained in Dulbecco's Modified Eagle Medium supplemented 10% fetal bovine serum. Cells were transfected by pNL[NlucP/ARE/Hygro] (#CS180902, Promega) and cells expressing the transgene stably were selected by hygromycin. The assay was performed according to the SOP of OECD TG442D.


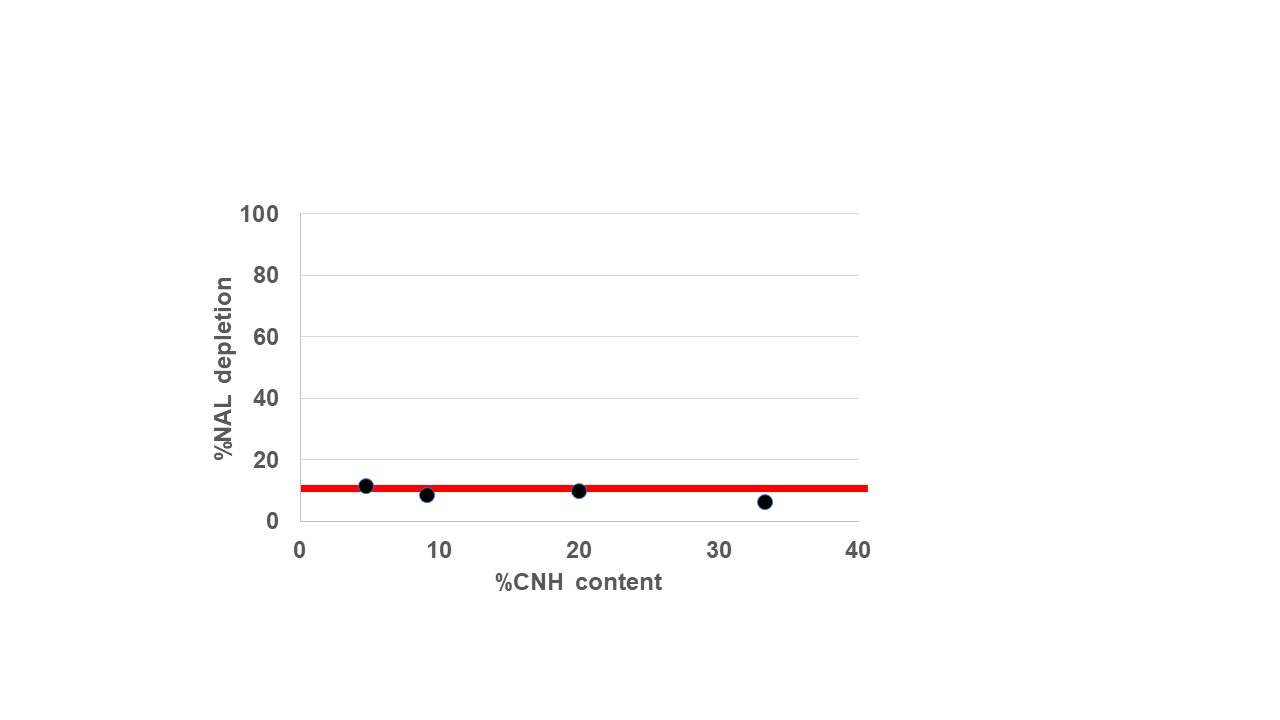


**Supplementary Figure S2**

The Cnh-cel sheets did not react with the test compound, N-acetyl lysine according to the assay evaluate skin sensitization AOP#1, OECD TG442C (4~11%> are the positive prediction lines, shown in red, respectively.). Note that the positive control, phenyl-acetaldehyde exhibited 88% depletion.

Supplementary methods:

The amino acid depletion reactivity assay kit was obtained from Fujifilm, Tokyo, Japan. The assay was performed according to the SOP of OECD TG442C and the manufacturer’s protocol.
